# Evaluation of the parasympathetic tone activity (PTA) for posttraumatic pain assessment in awake dogs before orthopaedic surgery - A prospective non-randomised clinical study

**DOI:** 10.1186/s12917-025-04561-x

**Published:** 2025-02-25

**Authors:** Eva Martha Billau, Max Bernhard Hubertus Weniger, Kathrin Büttner, Sabine Tacke, Eva Saskia Müller

**Affiliations:** 1 Small Animal Clinic (Surgery) Justus-Liebig-University, Giessen, Germany; 2 Unit for Biomathematics and Data Processing, Justus-Liebig-University, Giessen, Germany; 3 AniCura Ahlen GmbH Veterinary Specialist Centre for Small Animals, Ahlen, Germany; 4 Frankfurter-Strasse 114, 35392 Giessen, Germany

**Keywords:** Parasympathetic tone, Pain scoring, Posttraumatic dogs, Modified glasgow pain scale, Canine acute pain scale of the colorado state university

## Abstract

**Study design:**

A prospective non-randomised clinical study.

**Animals:**

A study group with 18 posttraumatic dogs before surgery and a control group with nine healthy dogs.

**Methods:**

Two different examiners evaluated the pain using two multidimensional pain scales (the Canine Acute Pain Scale of the Colorado State University (CSU-CAPS) and the Modified Glasgow Pain Scale (MGPS)) before the administration of methadone. During the administration of methadone, the Parasympathetic Tone Activity (PTA) was measured. In the control group, the PTA was measured without administration of methadone. In the statistical evaluation, correlation between PTA value and pain scores, and the predictive value of the PTA value in determining whether the animal was classified as painful was investigated. In addition, the results of the different pain scales and the results of the different examiners were compared.

**Results:**

The average PTA values of the control group were 45.67 (± 13.64). Two of nine (22.22%) animals in the control group have their average PTA value above the ‘pain-free state’ of 50. The average PTA values of the study group were 56.16 (± 15.11) and 51.05 (± 13.24) before and after methadone administration, respectively. Comparing the average values of the study group 30 s before methadone administration with the average values of the control group, there was no significant difference (*p* = 0.5403). Examiner A (experienced) classified 14 of 16 animals (87.5%) with the CSU-CAPS, and examiner A2 (inexperienced) classified 7 of 16 patients (43.75%) as painful. In 56.25% of the cases, both examiners (A and A2) reached the same decision when using CSU-CAPS. When using the MGPS, 10 of 18 patients (55.56%) reached the intervention level regardless of the examiner. In 88.89% of the cases, the two examiners reached the same decision; there is a highly positive correlation between the two examiners (Spearman correlation coefficient *rs* = 0.84). There was no correlation between the monitor and score values of both pain scales with either examiner.

**Conclusion:**

The PTA monitor on the awake animal was not suitable for pain detection. There were no statistically significant correlations of PTA scores with pain scale scores, regardless of the examiner. Similarly, the tendency for the study group to have lower PTA scores indicates that PTA also appears to be influenced by environmental factors.

## Introduction

Adequate analgesia and recognition of pain after surgical procedures is an important and growing issue in veterinary medicine. Just as important as analgesia in animals after surgery is analgesia in trauma patients before surgery. Initially after car accidents or other trauma, dogs and cats are usually in shock and get stabilised with infusion and analgesics until their general condition permits surgical treatment of fractures [1]. Patients must have an adequate analgesic protocol and must be evaluated regularly for pain severity during this time. Adequate analgesia must also be considered after surgical care, which can be reduced over time as pain subsides [2].

Nevertheless, inadequate analgesia is still common in patients with acute pain [2]. There are already many methods for assessing severity of pain and whether an animal needs analgesia based on the animal’s behaviour or facial expressions [3]. The accuracy of pain evaluation relies on the examiner’s experience, leading to potentially inaccurate recognition of pain [4, 5]. This is rather due to the different behaviour of the animals than the examiners individual assessment. While some dogs may show increased vocalisation when in pain, other dogs do not make any vocalisation even in severe pain [3].

To improve objectivity, it is helpful to use multidimensional established pain scales instead of unidimensional ones. These scoring systems include the behaviour of an animal in an attempt to estimate how much pain the patient is in [6, 7]. The functionality of these scales is difficult to validate because there is no adequate comparison or gold standard. There are many pain scales that have been evaluated for the different species, for acute and chronic pain, respectively. The Modified Glasgow Pain Scale (MGPS), which is used in this study is validated for acute pain [8]. In contrast, the Canine Acute Pain Scale of the Colorado Ctate University (CSU-CAPS) has not yet been validated.

In addition to analogue scales for pain evaluation by an examiner, there is the possibility to determine the Parasympathetic Tone Activity (PTA). An electronic device is used to draw conclusions about a painful stimulus and has been validated on unconscious patients [9–11]. The principle is based on the breath-dependent heart rate variability (HRV), which results from the vegetative influence at the sinus node of the heart. It measures the balance between sympathetic and parasympathetic tone which approximates nociception [12–14]. Nociception increases sympathetic tone with a corresponding decrease in parasympathetic tone. The heartbeat accelerates briefly in the inspiration phase and decreases in the expiration phase. There are various theories as to the exact underlying mechanism, but it is primarily assumed that this is mediated by the baroreflex [15]. Baroreceptors are predominantly located in the aortic arch or carotid sinus, where they serve as proportional-differential sensors that do not measure blood pressure as an absolute value but perceive pressure fluctuations and can also measure the rate of pressure rise. When these baroreceptors register an increase in pressure caused by respiratory activity during expiration in the thorax, the sympathetic nervous system is inhibited, and the parasympathetic nervous system is activated. The result of this process is a decrease in arterial peripheral resistance and heart rate. Other mechanisms, such as the stimulation of stretch receptors in the lungs, also appear to be involved. It is assumed that respiratory sinus arrhythmia leads to increased efficiency in gas exchange. During inspiration, perfusion is adapted to alveolar ventilation, while unnecessary heartbeats are suppressed during expiration to save energy [16].

To calculate the PTA, an electrocardiogram (ECG) is recorded and the distances between the individual R-spikes of each QRS complex are first measured. These R-R intervals are represented as frequencies by spectral analysis and can thus be divided into frequency ranges that make it possible to identify periodic patterns of HRV [17]. This makes it possible to divide HRV into three different main spectral components. One is the ‘very low frequency’ (VLF) component, which is relatively irrelevant to us and involves very low frequencies in the range of 20–50 mHz. The VLF component is subject to influences of thermoregulation, the level of circulating hormones such as catecholamines or the influence of the renin–angiotensin system. Next, there are low frequencies (LF) from 0.004 to 0.15 Hz, which reflect an activation of the sympathetic and parasympathetic nervous systems. Lastly, the high frequencies (HF) from 0.15 to 0.5 Hz are associated with a dominance of parasympathetic activity and are influenced by respiratory sinus arrhythmia. The real-time ECG normalises the calculated R-R intervals to 8 Hz and plots them on a 64-second graph. The influences of the patient’s baseline heart rate are removed by subtracting the mean M of the R-R intervals of the window at each sampling. The area under the curve (AUC) is calculated in four sections, with each section covering a period of 16 s. To be independent of the influence of respiratory rate, maxima and minima of the curves are determined, and the delineation of the outlines of the upper and lower areas is included in calculating the areas A1, A2, A3 and A4.

The PTA is calculated from a formula in which the minimum AUC from A1 to A4 is included [18]. The PTA is given as a value between zero and 100. A value of 100 corresponds to the highest proportion of parasympathetic-influenced respiratory sinus arrhythmia, and a value of zero corresponds to the lowest parasympathetic proportion with a low HRV [14]. In dogs, values below 40 are defined as severe pain and values in the range of 40–50 are defined as pain; values above 50 indicate a pain-free state [18]. A PTA value is calculated every second, and the immediate PTA (PTAi) and the mean PTA (PTAm) can be derived from this. The PTAi is calculated from the 54 previous values and displayed in yellow on the monitor. The PTAm is the averaged PTA index calculated from the 176 previous values and displayed in orange.

Several studies have shown that the PTA monitor can detect nociceptive stimuli in anaesthetised dogs and pigs during surgical procedures [9–11, 19–21]. The anaesthesiologist usually recognises this by an increase in heart rate, respiratory rate, and blood pressure. The studies indicate that even when heart rate and blood pressure remain normal, it is possible to detect if the animal is in a painful state by PTA, which can sense even weaker nociceptive stimuli [22]. In the study by Mansour et al. (2017), a significant drop in the PTA index occurred one minute after the predefined time points TClamp, TCut and TPrePTA. At TClamp clamps were placed in the skin, at TCut the surgical incision was made, and the TPrePTA was the point five minutes before a haemodynamic response occurred. A haemodynamic response was defined in this study as a 20% increase in heart rate and/or blood pressure. In this study the PTA is useful in anaesthetised dogs to detect pain even before a haemodynamic response [10]. The latest veterinary study by Mansour et al. (2021) compares PTA with mean arterial blood pressure in anaesthetised horses. One group was undergoing elective surgery, and the other group was surgery for colic. The authors of this study concluded that the animal’s health status must influence the PTA index since the horses in the ‘colic group’ had significantly lower values, which may be related to a predominance of sympathetic activity in situations of great stress. Furthermore, fluctuations in mean arterial blood pressure seem to be closely associated with fluctuations in PTA values, regardless of the horse’s health status [20].

In human medicine, the PTA corresponds to the Analgesia Nociception Index (ANI), which is already frequently used there to determine intraoperative and early postoperative haemodynamic changes due to pain [23]. It seems difficult to use the ANI to recognise pain in awake individuals. Gall et al. compared the ANI with the FLACC scale in children, where FLACC stands for ‘Face, Legs, Activity, Cry and Consolability’. This pain scale is used in human medicine to evaluate pain in children with mild to severe cognitive impairment. One result of this study is that the ANI is more suitable as a screening tool for the detection of postoperative pain. Patients in the control group (children without surgery) tend to have higher ANI values than the patients who underwent a surgical procedure. Conversely some patients in the control group had low ANI values [24].

In contrast to human medicine all previous veterinary studies have been conducted in anaesthetised animals. Therefore, this study will be the first using the monitor in awake patients, with the potential to demonstrate a new pain assessment. This could help to identify patients with pain who are not classified as having pain on conventional pain scales. Our hypothesis was that the awake animal is exposed to too many exogenous stimuli impairing pain detection by the PTA monitor.

## Methods

Prospective non-randomised clinical trial. This study included 18 dogs from the patient population of the Clinic for Small Animals (Surgery) of the Justus-Liebig-University in Giessen and nine dogs from employees of the same clinic in the context of a health check-up. The owners were informed, and their consent was obtained. Dogs under one year of age and over 12 years of age, brachycephalic dog breeds, and dogs suffering from cardiac arrhythmias and chronic nervous system degenerative diseases were excluded. Patients who received anticholinergics or drugs other than opioids, NSAIDs, and maropitant as part of the stabilisation protocol after trauma were also excluded. Fractious temperament also made measurements impossible and led to exclusion from the study.

The measurements of the control group were conducted as part of a voluntary health check-up. These dogs had no previous history of a painful condition and had not received any analgesic treatment in the preceding six months. All nine dogs received a general clinical examination, so only clinically healthy dogs were included in the control group. After that, the ECG leads of the monitor were connected to the patient. The red ECG electrode is clamped on the right, the yellow electrode on the left side on the chest directly behind the olecranon. The black electrode is clamped on the skin of the left knee crease. The dogs are free to sit, lie down, or remain standing. The electrodes are moistened slightly with alcohol until a good signal is obtained. Once the monitor obtained a good signal, the ECG was left for eight minutes and then removed. The dogs first had three minutes to get used to the situation, and the values of the remaining five minutes were included in the study.

**Fig. 1:**
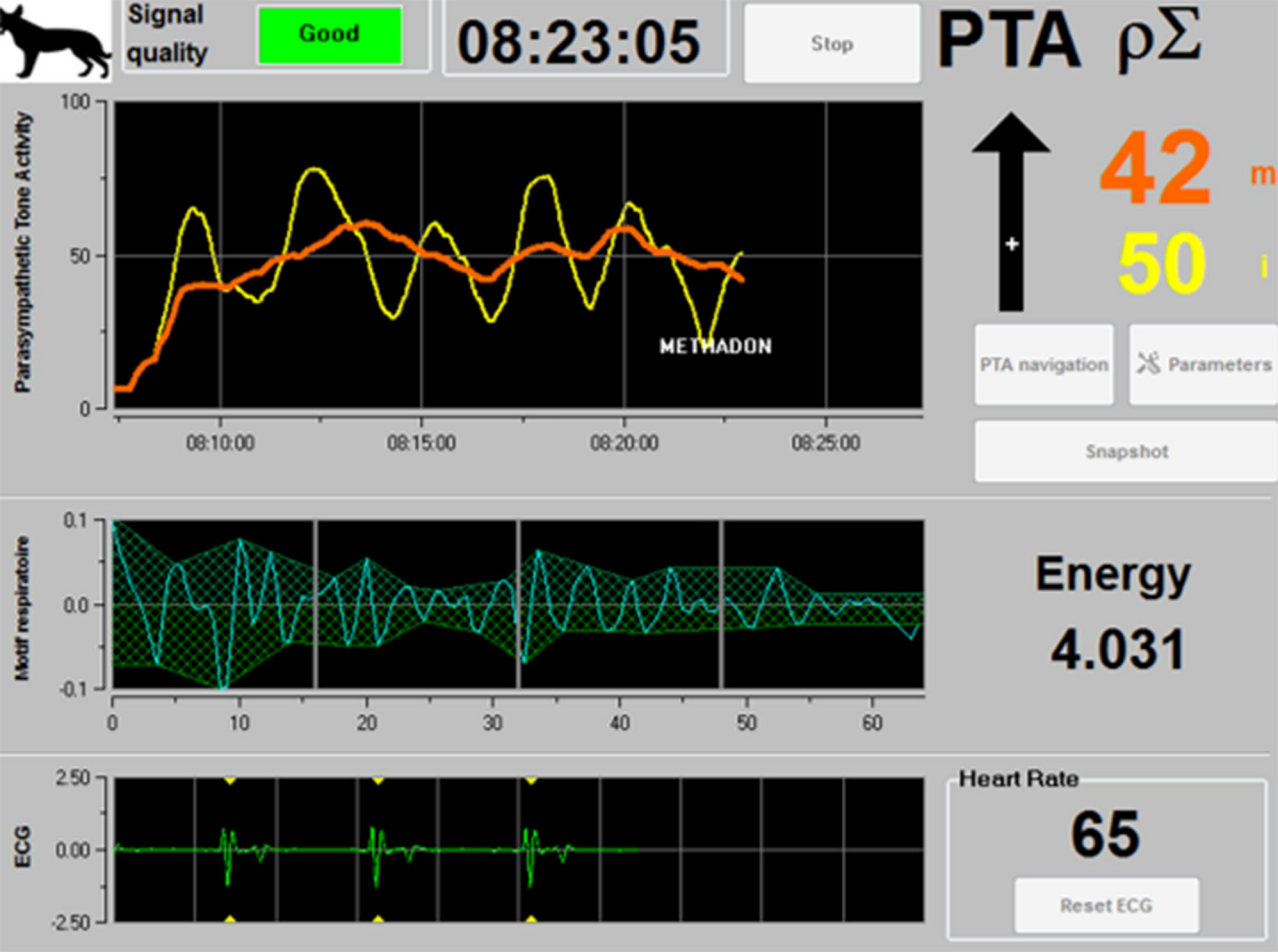
Screenshot of the PTA monitor with good signal quality. The orange curve represents PTAm and the yellow curve represents PTAi. The administration of methadone was marked as an event with ‘Methadone’

The study group included dogs that had sustained trauma with one or more fractures requiring surgical treatment after initial stabilisation. As part of the initial stabilisation phase, patients were administered only an analgesic (methadone), an antiemetic (maropitant) and infusion therapy. Additional treatment included oxygen flow by and, in a few cases, active warming. As in the control group, a general clinical examination was performed before the measurement on the awake animal and before the surgical intervention. According to the American Society of Anaesthesiologists, all patients with moderate systemic disease are classified as ASA class III [25]. Based on the pre-report trauma, all patients were classified as ASA class III after initial stabilisation. Dogs, after trauma, prior to surgical care, were evaluated using the Acute Pain Scale of Colorado State University (CSU-CAPS) and the Modified Glasgow Pain Scale (MGPS) [26, 27]. This was done 20 min before the regular administration of methadone (Comfortan^®^ 10 mg/ml Dechra Veterinary Products Deutschland GmbH, Aulendorf). The evaluations were always performed by two persons per patient, first by the head of the study, who is familiar with the pain scales and the evaluation of pain, and then by various veterinary colleagues in surgery, veterinary assistants, or veterinary medicine students. It was left to the examiner to decide which pain scale to use to begin the patient’s assessment. The monitor was then connected in the same way as in the control group. The dogs are free to sit, lie down, or remain standing. After acclimatising the patient to the situation, the monitor was left on the patient for three minutes. Then, 0.2 mg/kg methadone (Comfortan^®^) was administered intravenously, which was noted in the monitor as an event. After administration of the opioid, the PTA was recorded for an additional five minutes. Figure 1 shows a screenshot of the monitor as it appears when the signal quality is good.

Data evaluation was performed in the Unit for Biomathematics and Data Processing of the Department of Veterinary Medicine at the Justus-Liebig-University in Giessen. Statistical analysis was performed using the statistical programme SAS 9.4 (SAS^®^ Institute Inc., 2013).

To compare the results of the different pain scales by the two groups of examiners, the results are presented in four-fold tables, and the extent to which a correlation exists was examined. The degrees of agreement are expressed as percentages (%). The Spearman correlation coefficient (*rs*) is calculated to compare the MGPS scores of the different examiners.

The PTAm values are averaged over a 30 s period prior to the administration of methadone and then compared with the averaged PTAm values in the following five minutes of the administration of the drug. The PTAm values of the control group are averaged over a five-minute period and then compared with the averaged values of the study group before methadone administration. To calculate differences for dependent samples, the connected samples were tested for normal distribution beforehand, and paired *t*-tests were performed afterwards. Results with a *p*-value of less than < 0.05 were considered statistically significant.

When comparing PTA scores to pain scales, PTA scores of less than 50 are considered ‘painful’ for statistical analysis, as the PTA index for adequate analgesia in dogs is 50–100 [10]. To examine whether the PTA monitor was suitable for predicting the evaluated scores of the different pain scales, a binary logistic regression was performed. The criteria were classified as y = 0 ‘yes, the animal is in pain’ and y = 1 ‘no, the animal is not in pain’. The values of the PTA monitor served as predictors here. In addition, ROC (Receiver Operating Characteristics) curves are created to compare the individual pain scales with the PTA values. ROC values provide information about the diagnostic quality of a diagnostic test. AUC values (area under the curve) of 0.5 represent the bisector and indicate that a test has no diagnostic quality.

## Results

The study group consisted of 18 dogs, while the control group consisted of nine dogs. In the control group, the data of all nine animals could be evaluated. In the study group, two of the CSU-CAPS scores were right on the border between ‘painful’ and ‘not painful’, so these patients were excluded, leaving a total of 16 patients to compare the CSUCAPS with the PTA monitor and the MGPS. The animals in the control group were between one and twelve years old; the mean age was 5.89 years (± 4.121). The animals in the study group were between one and twelve years old; the mean age was 3.65 years (± 2.74). The weights of the animals in the control group ranged from 18 to 30 kg, and the mean weight was 24.89 kg (± 4.01). The weights of the animals in the study group ranged from 11 to 42 kg, and the average weight was 25.27 kg (± 9.08). The control group consisted of six males (66.6%) and three females (33.3%); two of the females and two of the males were castrated. The study group comprised twelve male (60%) and eight female (40%) animals. Of the males, seven were castrated, and of the females, two were castrated (Table 1).

**Table 1:** Descriptive comparison of control and study group in terms of average age, weight, and gender distribution

	Age	weight	male	female
Control Group	5,89 years (± 4,121)	24,89 kg (± 4,01)	6 (66,6%)	3 (33,3%)
Study Group	3,65 years (± 2,74)	25,27 kg (± 9,08)	12 (60%)	8 (40%)

With the CSU-CAPS, examiner A (experienced) classified 14 of 16 animals (87.5%) and examiners A2 (inexperienced) classified 7 of 16 patients (43.75%) as painful. In 56.25% of the cases, both examiners (A and A2) reached the same decision when using CSU-CAPS (Table 2).

**Table 2:** Comparison of the different examiners (A and A2) when using the CSU-CAPS

Examiner A	Examiner A2		
	Painful	Not painful	Total
*Painful*	7 (43,75%)	7 (43,75%)	14 (87,5%)
*Not painful*	0	2 (12,5%)	2 (12,5%)
*Total*	7 (43,75%)	9(56,2%)	16 (100%)

When the MGPS was used, both examiners classified 10 of 18 patients (55.56%) as painful. In only two cases, the results of both examiners differed, which is why, in 88.89% of the cases, the two examiners reached the same decision for the MGPS (Table 3). When comparing the number of points given to each patient, there was a highly positive correlation between the two examiners (Spearman correlation coefficient *rs* = 0.84).

**Table 3:** Comparison of the different examiners (B and B2) when using the MGPS

Examiner B	Examiner B2		
	Painful	Not painful	Total
*Painful*	9 (50%)	1 (5,56%)	10 (55,56%)
*Not painful*	1 (5,56%)	7 (38,89%)	8 (44,44%)
*Total*	10 (55,56%)	8(44,44%)	18 (100%)

The average PTA values of the control group are 45.67 (± 13.64). The average PTA values of the study group were 56.16 (± 15.11) and 51.05 (± 13.24) before and after methadone administration, respectively (Table 4). Comparing the average values of the study group 30 s before methadone administration with the average values of the control group, there was no significant difference (*p* = 0.5403). If all PTA values above 50 represent a pain-free state, two of nine (22.22%) animals in the control group have their average PTA value above this limit.

If an average PTA value of less than 50 reflects pain, 7 of 18 dogs in the study group were below this threshold before methadone administration. After a paired-sample *t*-test, the mean monitor values 30 s before and five minutes after medication administration differ significantly (*p* = 0.0379).

**Table 4:** Parasympathetic tone activity in the control and study group

	Mean PTA	Standard deviation	Minimum PTA	Maximum PTA
Control Group	45.67	13.64	22.00	85.00
Study Group before methadone	56.16	15.11	29.00	77.00
Study Group after methadone	51.05	13.24	30.00	78.00

**Fig. 2:**
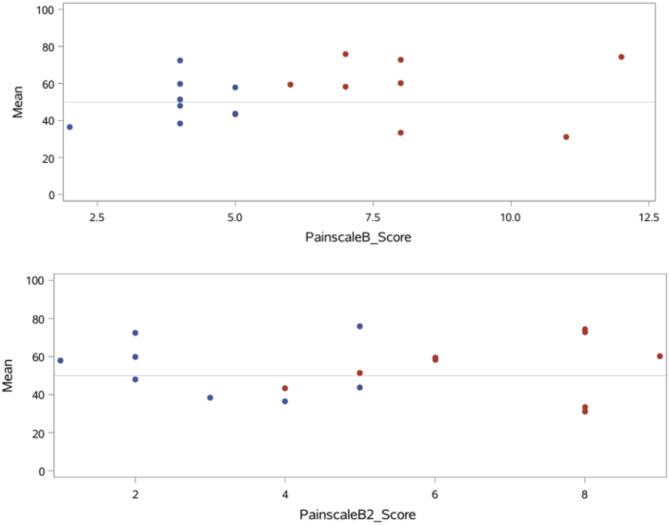
Comparison of the score values of the MGPS of observer B and B2 (x-axis) with the PTA values (y-axis) before methadone administration

**Fig. 3:**
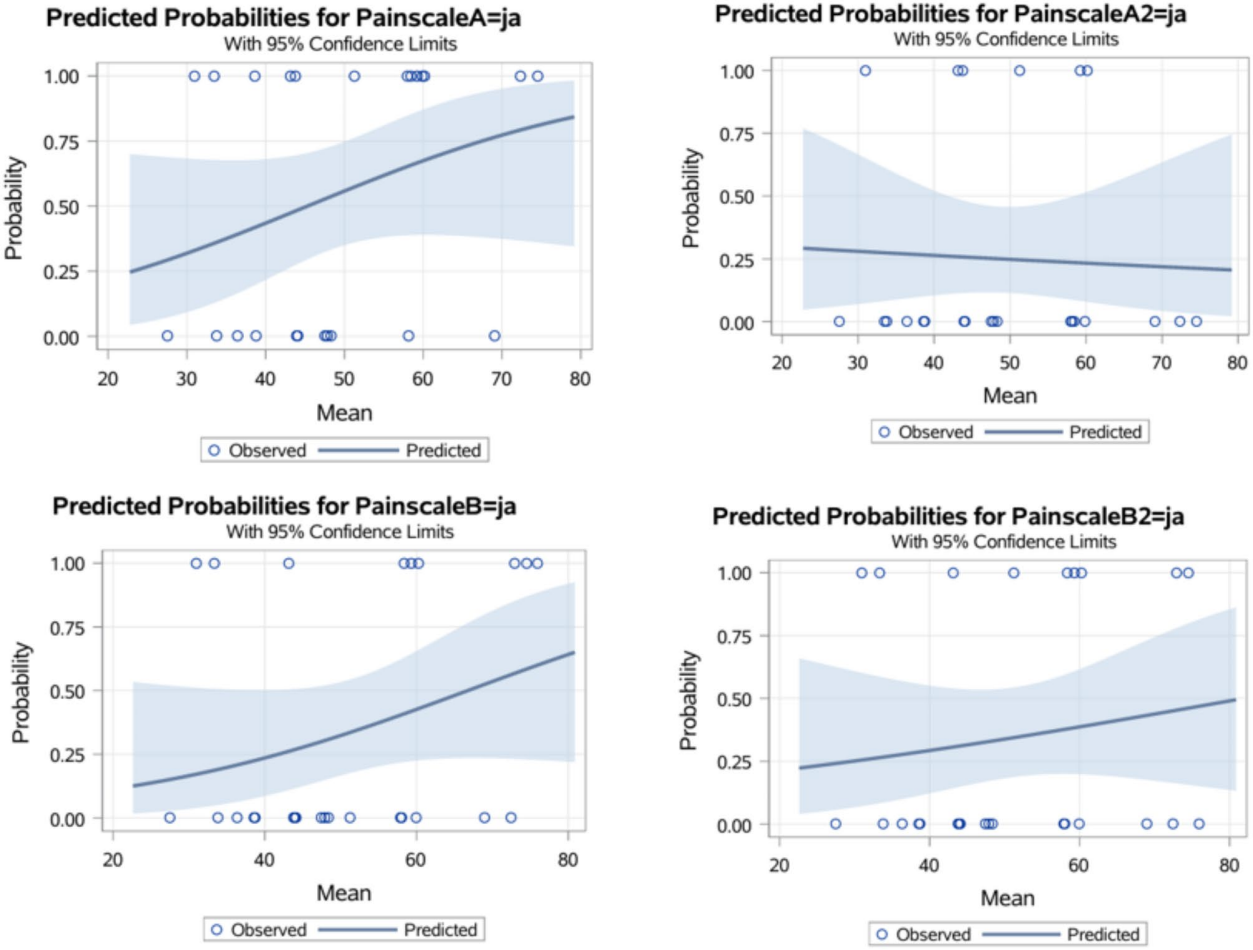
Binary logistic regression to visualise the predictive power of the monitor in relation to the results of the Pain Scales

**Fig. 4:**
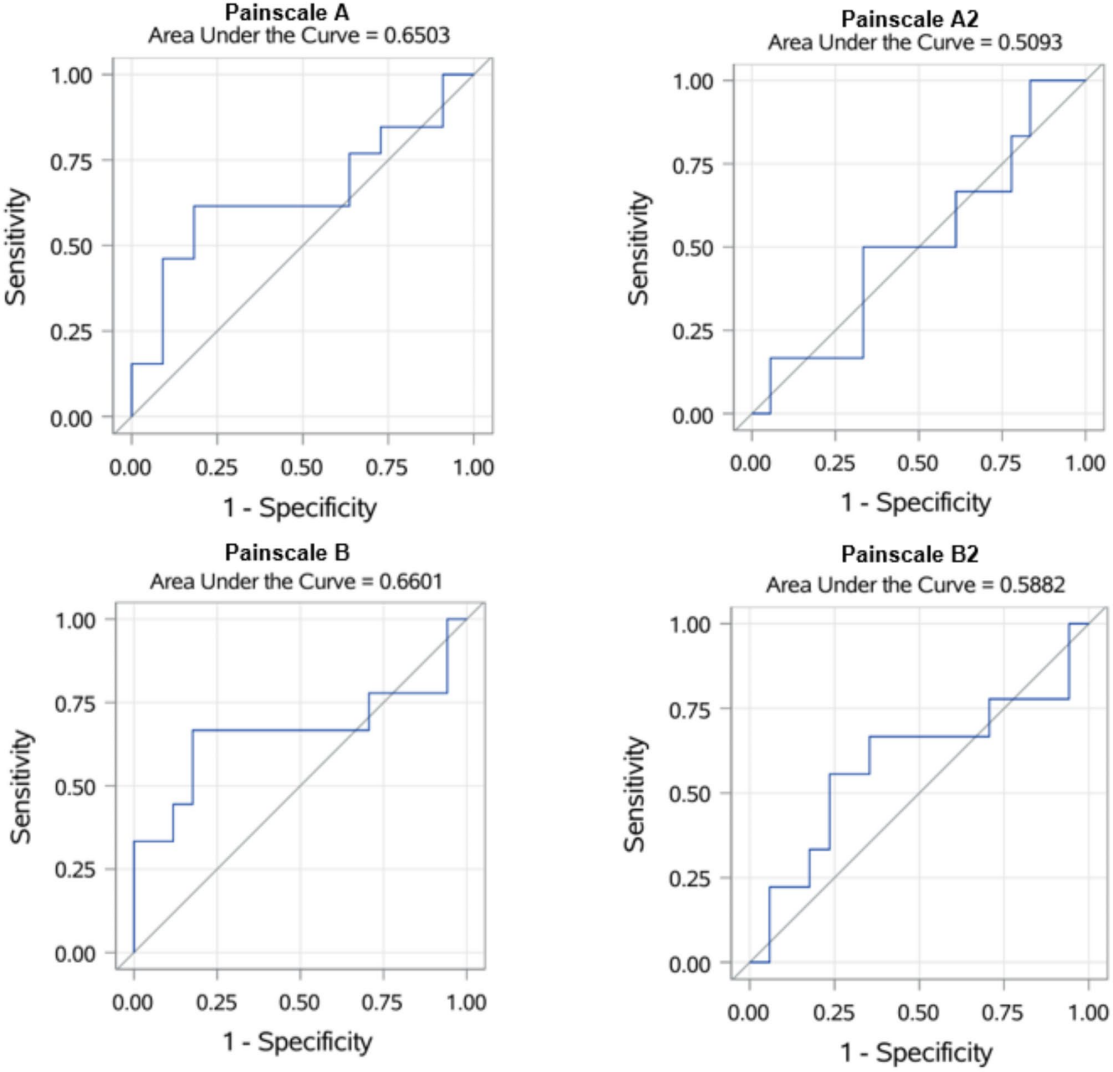
ROC curves of the logistic regression in relation to the individual pain scales

There was no statistical correlation between the PTA values and the score values of both pain scales in either examiner (Fig. 2). In the binary logistic regression, the monitor is also not suitable for predicting the results of the pain scales. Figure 3 shows the binary logistic regression for each pain scale. The x-axis shows the values of the predictor, in this case the PTA values from zero to 100. On the y-axis, a value of ‘zero’ means that the animal is not considered to be painful and a value of ‘one’ means that the animal is painful. The steeper the curve of the statistical regression, the more suitable the predictor is for predicting an event. It can be seen very clearly here that a somewhat steeper curve can only be seen for ‘Painscale A’, which corresponds to the evaluation of the CSU-CAPS of the experienced examiner. Otherwise, the logistic regression curves are very flat, which graphically illustrates the lack of predictive power of the PTA monitor. At least the ROC curves and corresponding AUC values for the comparison of the individual pain scales with the PTA values are shown in Fig. 4.

## Discussion

In this study, the main aim was to evaluate whether the PTA monitor was suitable for detecting pain in the awake animal. The results of this study indicate that the PTA monitor is not an effective tool for identifying pain in conscious animals, because many dogs in the control group were classified as painful. In the control group, the average PTA values are 45.67%. In the manual of MDoloris Medical Systems, the range for pain in anaesthetized dogs is between 40 and 50, and below 40, the manufacturer speaks of extreme pain [14]. On average, all animals in the control group are below the manufacturer’s ‘pain value’. Looking at the details, only two out of nine animals in the control group are above the threshold of 50. Since the dogs in the control group are healthy, pain-free dogs, it is most likely a wrong assessment of pain. In awake humans, there are no precise reference values in the literature that indicate a painful state. In the study of Issa et al. (2017), the PTA in awake, pain-free human patients is, on average, 82. Still, the values show a considerable scatter, and in humans, some patients show values of around 40, although no painful stimulus occurred at that time [28]. In Jess et al. (2016), the average values before any stimulation were 82.1 +/- 10.7 and showed a high dispersion [29]. The higher values observed in human medicine, could be explained by the fact that the measurement procedure can be precisely explained to people before the study. Simply connecting a foreign object, such as an electrocardiogram, can be associated with enormous stress for a dog. Since stress is known to affect our sympathetic nervous system, this may explain why the PTA values of the control group are in such low ranges.

Looking at the patients in the study group, the average PTA value before methadone administration is 56.16, which tends to be slightly higher than the PTA value of the control group. If the PTA were indicative to recognise pain, this value would have to be significantly lower. If the threshold value is assumed to be 50, 12 of the 18 patients are above this threshold value and would be pain-free according to the PTA index. In direct comparison, there is no significant difference (*p* = 0.5403) between the PTA values of the awake patients before methadone administration and the control group. A significant difference would have meant that the monitor could detect pain in the animal. One reason for the slightly higher values could be the residual effect of the last administration of methadone four hours before the measurement. Methadone has a slightly depressant effect on the central nervous system (CNS), thus reducing stress and sympathetic tone. In addition, it could play a role as patients after trauma are more likely to have higher vagotone or cardiac arrhythmias such as ventricular extrasystoles [30]. Ventricular extrasystoles did not occur in the patients in the study group, but the higher vagotone may influence the PTA measurement. Another explanation for the tendency of the values of the study group to be slightly higher may be that the patients are accustomed to the connection of an electrocardiogram due to the handling during the inpatient stay and are, therefore, less stressed than the animals of the control group.

Another striking feature is that PTA values are significantly lower after the administration of methadone than before (*p* = 0.0379). Several hypotheses can explain this fact. When administered intravenously, methadone acts immediately due to rapid redistribution from the blood plasma to the CNS, which directly exerts its analgesic effect [31]. Therefore, it can be assumed that the PTA values, which are measured in the three minutes after injection, are already influenced by the effect of methadone. Since parasympathetic activity is determined by the electrocardiogram, it is possible that the bradycardia induced by the opioid influences the PTA values [31]. In addition, PTA is dependent on respiratory sinus arrhythmia, which may also be affected by the opioid, as opioids can cause respiratory depression [31]. Müller (2021) notes in her study in anaesthetised dogs that the values of the PTA monitor three to five minutes after administering a drug, such as methadone, are not evaluable [21]. Consequently, in the present study, the values five to ten minutes after the methadone injection should have been compared with the values before the administration. However, this would have required leaving the monitor connected to the awake animal for a longer period, which is usually not tolerated by animals and makes the measurements impractical.

Theoretically, the MGPS scores should be inversely related to the PTA scores because the higher the pain score, the lower the PTA scores should be. However, looking at the results from both examiners, only 44.44% agreement between the MGPS and the PTA monitor was reached. The graphical representation comparing the assessment from both examiners in Fig. 2, shows the lack of correlation between the results. Comparing the results of the CSU-CAPS with the values of the PTA monitor, we found agreement in 37.5% of the cases for the experienced examiner and in 56.2% of the cases for the inexperienced examiner. This roughly mirrors the results of many studies in human medicine.

There was only 56.25% agreement between the two different examiner groups when evaluating pain using the CSU-CAPS. In contrast, there was 88.89% agreement when evaluating the MGPS. Seven out of 16 patients (43.75%) were classified as ‘pain-free’ according to the CSU-CAPS by the untrained examiners, which were considered ‘painful’ by the trained examiner. It can be inferred that if inexperienced examiners used CSU-CAPS, patients in pain would likely not be noticed, and they would then not receive the necessary analgesia. Cerny (2011) compared the CSU-CAPS with a ‘Dynamic and Interactive Visual Analogue Scale’ (DIVAS) in cats and concluded that a large variability prevails between the different examiner groups as well as within the groups themselves [5]. It seems that MGPS can be better integrated into daily clinical practice since it cannot always be guaranteed that pain evaluation is only performed by experienced examiners. This theory is supported by a study from the Netherlands that investigated the use of MGPS in clinical practice and confirmed its practicality for detecting pain [8]. However, this study did not indicate whether patients classified as non-painful were indeed pain-free. This is because there is still no gold standard in pain evaluation against which to measure pain scales. The high discrepancy between the two examiners comes from the fact that by using the MGPS the experienced examiner classifies only 55.56% of the animals as painful, and when using the CSU-CAPS 87.5%. In the group of inexperienced examiners, the same percentage of patients were classified as painful when using MGPS, but only 43.75% when using CSU-CAPS. Thus, the inexperienced examiner arrived at the same assessment in 75% of the cases when using both pain scales on the same patient, but the experienced examiner only in 62.5%. This result suggests that the MGPS may not detect some painful patients by both examiners because they remain below the intervention level. When evaluated by the MGPS, only about half (55.56%) of the patients reached the intervention level and would thus receive an analgesic according to this pain scale. A closer look at the individual questions and the answer options reveals that a patient is more likely to reach the intervention level if it interacts with the examiner through vocalisation. However, this does not apply to all patients, as dogs do not show their pain only through vocalisation [3]. Signs of pain can be very subtle and may only be reflected in a change in facial expression, which is why ‘grimace scales’ have become important in the context of pain evaluation [32–34]. This leads to the assumption that if the MGPS pain scale alone is used, many painful patients will remain below the intervention level despite severe pain and will not receive an analgesic. This is because if one looks at the pain scale results from Colorado State University, the experienced examiner concludes that the patient is in a painful state 87.5% of the time. The CSU-CAPS includes many more parameters based on the patient’s facial expressions and gestures. This can be challenging for the inexperienced examiner and explains the high discrepancy between the two examiners. Unfortunately, there is still no gold standard against which to measure both pain scales. However, it is striking that by MGPS, only 55.56% of patients are in pain, even though they are all, one-day post major trauma, and the last analgesic administration was four hours ago. Further or other pain scale methods should be considered, because analgesics are still underused in many cases. Simon et al. name this problem ‘oligoanalgesia’ and advocate for putting more focus on evaluating pain in college and educating staff in clinical practice [2].

In human medicine, there are studies in awake patients that compare the Analgesia Nociception Index (ANI) scores with unidimensional pain scales such as the Numeric Rating Scale (NRS). They concluded that there is a correlation between the two pain evaluation methods in the immediate postoperative period [35, 36]. This would be a helpful complementary tool for pain evaluation, especially for young children or cognitively impaired individuals who cannot self-assess their pain on an NRS. The Face-Legs-Activity-Cry-Consolability (FLACC) scale is often used for pain evaluation in young children. The ANI in children measured immediately postoperatively correlates with the values on the FLACC scale [24]. Ledowski et al. (2013) recognised a low correlation between the ANI and the NRS but concluded in their study that there was only a low sensitivity and specificity [37]. The study by Charier et al. (2019) compared the ANI and the Variation Coefficient of Pupillary Diameter (VCPD) with a Visual Analogue Scale (VAS), pupillary diameter (PD), and pupillary light reflex (PLR). They concluded that the VCPD correlated more with the VAS than the ANI in the postoperative period, and the PD and PLR had no correlation with the VAS [38]. They discussed many reasons for this lack of correlation, such as respiratory depression from anaesthesia, which could affect the ANI. This does not play a role in the dogs in this study, as they were evaluated before general anaesthesia. Similarly, Charier et al. (2019) cite that for evaluating pain, looking at the pupil has various limitations, as the size of the pupil is dependent on both opioids and the exposure in the room, and therefore, the VCPD is more appropriate than the static parameters PD and PLR [38]. Only two studies, which took place before general anaesthesia and evaluated healthy subjects, are known to the author. In one study, 23 participants were given nociceptive stimuli in the form of small electrical pulses while the ANI was recorded, and they had to rank their pain on an NRS. In this study, only a very weak negative correlation between the ANI and the scores on the NRS was found. They compared the ANI values with changes in ANI (ΔANI). The ΔANI had a slightly higher negative correlation, but no clear negative correlation was seen [28]. The study by Jess et al. (2016) differed in that patients were not continuously subjected to increasingly painful electrical stimuli but did not know whether the stimulus would be painful or non-painful. Nonetheless, the study came to the same conclusion, namely that the ANI has no negative correlation with an NRS and is likely to be strongly influenced by stress and emotion [29].

The present study has several limitations. It is possible that the administration of methadone four hours before the assessment with the pain scales and the PTA monitor influenced the results due to some residual effect. To eliminate this factor, patients would have to be assessed before receiving any pain medication and the measurement would have to be made by the monitor. This was rejected by us for ethical reasons, as patients need to receive an opioid as soon as possible after a major trauma. We assumed that the patients were painful due to their illnesses and concluded that it may not be possible to identify some patients as painful with the help of MGPS. It is possible that some patients did not actually show any clear pain due to the residual effect of methadone. However, this does not explain the fact that more patients were classified as painful by the CSU-CAPS. Additionally the sample size was small in the control and study group and the study was not blinded. Follow-up studies with a larger patient group could potentially increase statistical significance. One possibility would be to evaluate using PTA in dogs in the immediate postoperative period when patients are still lightly sedated from general anaesthesia and not stressed by environmental factors. However, the drugs administered during general anaesthesia would be an unavoidable factor affecting the measurement.

To conclude, the PTA monitor is not an effective tool for detecting pain in awake dogs. The study also demonstrates that there is a high degree of variability between individual examiner groups when utilising the CSU-CAPS, which is not reproduced when using the MGPS. Finally, despite the use of established pain scales, it appears that some dogs cannot be identified as being in pain especially when the examiner is not used to pain recognition.

## Conclusion

In summary, pain evaluation using multidimensional pain scales in the awake dog shows no correlation with PTA, mirroring the results of studies from human medicine in awake subjects. Because pain recognition remains challenging, evaluating pain regularly and educating staff is important.

## Data Availability

The datasets used and analysed during the current study are available from the corresponding author upon reasonable request.

## Abbreviations

ANI: Analgesia Nociception Index
ASA: American Society of Anaesthesiologists
CSU-CAPS: Colorado State University– Canine Acute Pain Scale
ECG: Electrocardiogram
FLACC Scale: Face, Legs, Activity, Cry, and Consolability– Scale
HRV: Heart rate variability
MGPS: Modified Glasgow Pain Scale
PD: Pupillary diameter
PLR: Pupillary light reflex
PTA: Parasympathetic Tone Activity
PTAi: PTA instantaneous
PTAm: PTA mean
VAS: Visual Analogue Scale
VCPD: Variation Coefficient of Pupillary Diameter
